# Experience with an OSCE anamnesis station via Zoom: Feasibility, acceptance and challenges from the perspective of students, simulated patients and examiners during the COVID-19 pandemic

**DOI:** 10.3205/zma001565

**Published:** 2022-09-15

**Authors:** Stephanie Herbstreit, Sven Benson, Carina Raiser, Cynthia Szalai, Angelika Fritz, Frederike Rademacher, Gertraud Gradl-Dietsch

**Affiliations:** 1 Universitätsklinikum Essen, Klinik für Unfall-, Hand- und Wiederherstellungschirurgie, Essen, Germany; 2 Universität Duisburg-Essen, Medizinische Fakultät, Institut für Didaktik und curriculare Entwicklung in der Medizin, Essen, Germany; 3 LVR-Klinikum Essen, Klinik für Psychiatrie, Psychosomatik und Psychotherapie des Kindes- und Jugendalters, Essen, Germany; 4 Universitätsklinikum Essen, Klinik für Anästhesiologie und Intensivmedizin, Essen, Germany; 5 Universität Duisburg-Essen, Medizinische Fakultät, Simulations-Patienten-Programm, Essen, Germany

**Keywords:** online assessment, OSCE, communication, assessment development, telemedicine

## Abstract

**Aim: **Assessments of practical clinical competencies pose a challenge during the COVID-19 pandemic. Reports about OSCE stations administered online show that, despite technical feasibility and acceptance, there is a lingering desire for in-person assessments. Barriers and challenges must therefore also be identified in regard to the future integration of digital competencies into the curriculum. Based on a study investigating the feasibility and acceptance of an online OSCE anamnesis station and the descriptions given by students, simulated patients and examiners of the challenges and limitations, we make recommendations for necessary future adaptations to anamnesis training and testing in the context of telemedicine.

**Method: **We surveyed students after completion of an OSCE anamnesis station, adapted to the telemedical setting, that was administered as an alternative assessment to 149 students via Zoom^®^. Using semi-structured interviews, we analyzed the resulting challenges and limitations as seen by all of the participants.

**Results: **We confirm the existence of good technical and organizational feasibility, positive learning experiences through feedback, the acquisition of clinical competencies, and a high acceptance of this format as an alternative assessment during the pandemic. Using the semi-structured interviews, it was also possible to analyze additional categories that identify necessary adaptations of this type of format.

**Conclusion: **Adaptation of the content-based training for all of the participants and a targeted revision of the checklists, e.g., regarding communication techniques in a telemedicine setting, is required due to the effects of the online format on communication and interactions between students and simulated patients.

## Zusammenfassung

**Zielsetzung: **Leistungsüberprüfungen klinisch-praktischer Kompetenzen sind im Rahmen der COVID-19-Pandemie eine Herausforderung. Berichte über online durchgeführte OSCE-Stationen zeigen, trotz technischer Machbarkeit und Akzeptanz, den bleibenden Wunsch nach Präsenzprüfungen. Barrieren und Herausforderungen müssen daher auch im Hinblick auf die zukünftige Integration digitaler Kompetenzen in das Curriculum identifiziert werden. Mit Hilfe einer Untersuchung zur Machbarkeit und Akzeptanz bei der Durchführung einer Anamnese-OSCE-Station im Online-Format und Darstellung von Herausforderungen und Limitationen von Seiten der Studierende, der Simulationspatient*innen und der Prüfenden benennen wir Empfehlungen für zukünftig notwendige Anpassungen für Anamnese-Schulungen und Prüfungen unter telemedizinischen Aspekten.

**Methodik:** Im Anschluss an eine als Prüfungsersatz mit 149 Studierenden via Zoom^®^ durchgeführten und auf ein telemedizinisches Setting angepasste Anamnese-OSCE-Station, führten wir eine Fragebogenerhebung bei Studierenden durch. Mit Hilfe von semistrukturierten Interviews analysierten wir die sich daraus ergebenen Herausforderungen und Limitationen von Seiten aller Beteiligten.

**Ergebnisse: **Wir konnten eine gute technische und organisatorische Machbarkeit, positive Lernerfahrungen durch Feedback und den Erwerb von klinischen Kompetenzen, sowie eine hohe Akzeptanz des Formates als Prüfungsersatz im Rahmen der Pandemie bestätigen. Mit Hilfe der semistrukturierten Interviews konnten zusätzlich Kategorien analysiert werden, die notwendige Anpassungen eines solchen Formates identifizieren.

**Schlussfolgerung: **Eine durch das Online-Format veränderte Kommunikation und Interaktion zwischen Studierenden und Simulationspatient*innen bedarf einer Adaptation der inhaltlichen Schulung aller Beteiligten und einer gezielten Anpassung der Checklisten, z.B. bezüglich Kommunikations-Techniken für ein telemedizinisches Szenario

## 1. Introduction

The education of future physicians is challenged by the COVID-19 pandemic – and also beyond it – in regard to teaching and testing competencies. Assessments to test abilities remain indispensable during the pandemic and require appropriate adjustments [1], [2], [3]. The Objective Structured Clinical Examination (OSCE), a widely used method to assess practical clinical skills [4], poses a particular challenge in the context of the pandemic [5], [6]. Prior to the pandemic, there were already reports of positive experiences with online OSCEs that were offered, for example, to accommodate large geographic distances [7], [8]. Standardized tele-OSCE simulations were piloted also for the purpose of integrating telemedicine into the curriculum and to identify barriers and challenges, but without resulting in recommendations for any clear adjustments [9], [10], [11], [12]. During the COVID-19 pandemic, different online OSCEs were piloted and evaluated positively. The use of different technologies was recommended for this when administering online or virtual OSCEs [13], [14]. The results showed good feasibility [15], [16], [17], [18], [19], [20] and positive learning experiences [5], [21], [22]. In particular, technical and organizational recommendations were addressed, but necessary adaptations based on telemedical aspects have not yet been commented on in a critical, game-changing way [5], [16], [18], [23]. As the use of online OSCEs is being increasingly discussed in the context of the pending digitalization process [14], [24], [25], [26], [27], detailed feedback from the main participants is needed in order to develop this format further [28], [29].

At the University of Duisburg-Essen Medical School, basic competencies, and particularly anamnesis, are taught in the first clinical semester. The acquired skills in anamnesis, as the central shared learning objective of the participating subject areas, were to be assessed under pandemic conditions, which is why an OSCE in an online format was developed.

The aim of this study was to gain information about any necessary adaptations for future online OSCE anamnesis stations in a telemedicine setting.

## 2. Material and method

Applying a mixed-methods approach, information on the feasibility and acceptance of an online OSCE anamnesis station was gathered from students by means of a questionnaire. Using semi-structured interviews, the challenges and limitations were identified from the perspective of the students, simulated patients (SPs) and examiners.

### 2.1. Assessment procedure

**Figure 1:**
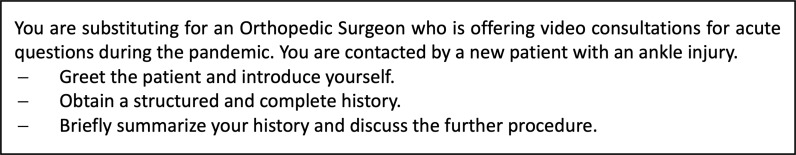
Examination instruction for the students, published in the chat.

A total of 149 students participated in an OSCE anamnesis station which was administered during the 2020/21 winter semester via Zoom^®^. An anamnesis station that had already been proven in regular OSCE assessments was used, and SPs already familiar with the roles were assigned to it. Figure 1 (Fig. 1) contains the instructions for the simulated medical consultation, adapted for a telemedicine scenario, as well as the assessment task to make the virtual case history taking as realistic as possible. The evaluation items on the assessment checklist were left unchanged.

**Figure 2:**
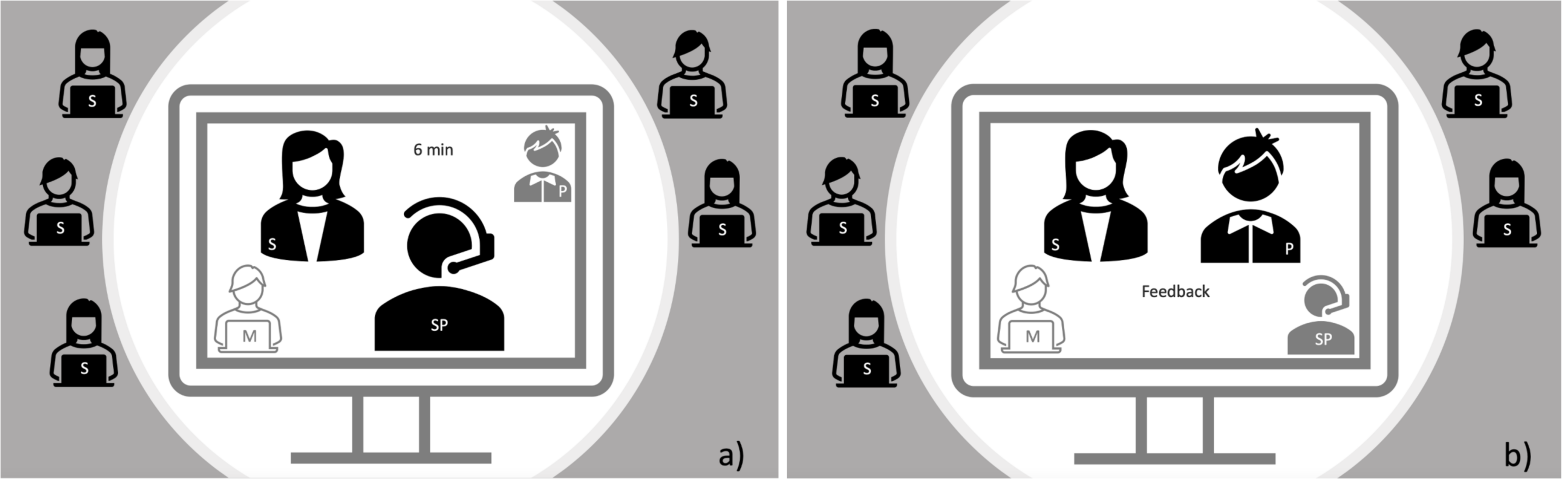
Anamnesis-OSCE station via zoom with moderators (M), simulation sponsors (SP), students (S) and examiners (P). a) Examination situation, b) feedback situation (grayed out persons are not visible with video in the situation. Students outside the circle are in the waiting area of the video conference).

The students received structured instructions about the assessment procedure by email. Each student was sent a personalized Zoom-link time slot for the assessment. For the online assessment, multiple blocks of 1½ hours each were set up in 3 parallel Zoom rooms over a period of 4 days. Six students were scheduled for each assessment block. The assessment duration was 6 minutes plus 4 minutes of feedback, just as before the pandemic. At the start of each assessment block, the students were given an introduction by the moderators and examiners. One examinee remained in the room while the other 5 were sent to a waiting room from which they were brought separately, one after the other, into the assessment room. The assessment instructions were given in the chat. Figure 2 (Fig. 2), point a depicts the precise assessment situation with invisible examiners and visible SP. Figure 2 (Fig. 2), point b depicts the configuration during the structured feedback with the visible examiner and invisible SPs. No scores were given.

#### 2.2. Student questionnaire

Access to the web-based questionnaire was sent by email to all of the participating students (N=149). The survey was conducted using the open-source software Sosci Survey [https://www.soscisurvey.de/]. Participation was voluntary and anonymous. All of the participants gave their consent at the beginning of the online survey. Approval was given by the responsible ethics commission (no. 20-9202-BO). In addition to collecting information on age and gender, the questionnaire covered a total of 22 items (see table 1 (Tab. 1)). The items were worded based on studies in which the feasibility and challenges of online OSCEs had been investigated before and during the pandemic [15], [30].

What was captured was an estimation of the organizational and technical feasibility. The acceptance of the format was recorded with the questions about the overall evaluation, the suitability of the format as an assessment and as a learning experience. Furthermore, questions were asked about the experience of interacting with the SPs and the authenticity. The evaluation was done using a six-point Likert scale (see table 1 (Tab. 1)). The descriptive analysis of the survey data was performed using SPSS software (version 27). The items were presented as median with 25^th^ and 75^th^ percentiles and as mean value±standard deviation.

#### 2.3. Semi-structured interviews of students

The primary goal of the semi-structured student interviews was to gain a deeper understanding of the items that showed a pronounced heterogeneity in the responses. A total of eight students were interviewed via Zoom in two groups of four students each. The invitation to be interviewed was sent by randomized email. The following topics were followed up on in more detail using a semi-structured interview guideline:

- nervousness/anxiety,
- pros and cons of an online test,
- influence of the online format on the interaction,
- suitability as a testing format versus as a learning experience,
- recommendations.

The analysis was carried out using structured qualitative content analysis [31], [32] and was descriptively analyzed according to category. The statements in each category were paraphrased and summarized as quintessential core statements. These core statements were then each assigned an anchor statement, which was quantified in terms of frequency.

#### 2.4. Semi-structured interviews – simulated patients and examiners

Individual semi-structured interviews based on a guideline were held via Zoom with the participating SPs (N=6) and examiners (N=6). Since the literature does not yet contain any analysis of the experiences of all of the participating groups, the questions for the interview guideline were developed in deductive categories based on an analysis of the existing literature on online OSCEs. The actors were requested to give* general feedback on the situation for taking a case history*. In addition, they were asked to assess the* authenticity of the situation surrounding the medical consultation* and about *problems*. In terms of their experiences with in-person assessments, they were asked if the students had interacted with them differently than otherwise during the virtual OSCE and what impressions they had regarding the *nervousness of the students* compared to in a face-to-face assessment. The analysis of the interviews was done using summarizing qualitative content analysis [31], [32]. Frequently mentioned statements and unusual statements were presented as interpretations and, in part, cited verbatim.

## 3. Results

### 3.1. Questionnaires

N=116 students (response rate: 78%) filled out the questionnaire, of which 81 (69,8%) were female and 35 (30,2%) male (see table 1 (Tab. 1)). A total of 35% of the students stated that they had prior experience in a healthcare profession. The majority of the students reported that they felt well prepared organizationally for the assessment. They were only a little anxious, whereby the analysis of the responses to the question about nervousness showed a wide range. The experience of the assessment was predominantly rated positively and the students stated that they generally felt comfortable with the situation. Despite this, the follow-up questioning on whether an in-person assessment would have been preferable to them showed a nonuniform picture. The virtual meeting with the SPs was evaluated to be mostly realistic. A wide distribution was also seen with regard to the question if the online format influenced the interaction between students and SPs. The majority of the students found that the case history taking was really not difficult in terms of content. The feedback from the examiners was valued by the vast majority, and the students stated that they had felt rather competent during the assessment. While taking the case history did help the students to reflect on and improve their own techniques, the opinions on whether the online format is suitable for assessing anamnesis skills were widely distributed, ranging from agreement to rejection. The question whether the online format is a suitable method to assess verbal communication skills was more often answered in the affirmative. In contrast, the assessment of nonverbal communication skills in an online format was viewed more critically. The format's suitability as a practical exercise for interacting with “real” patients was also seen more critically. Despite this, the format was evaluated as being a mostly fair assessment method and seen as having no technical challenges.

#### 3.2. Student interviews

The items with heterogeneous response patterns were followed up on using structured interviews (N=8 students). The responses are presented as inductive categories, and anchor statements are cited as examples with information about the frequency of an aspect (NS=X), (see attachment 1 ).

##### 3.2.1. Nervousness/anxiety

Uncertainty was expressed specifically in relation to the unknown reason for the consultation with the SPs. The low level of anxiety was explained as a consequence of being in a safe assessment situation and having a low level of exam stress at the time. Concerns about their own competence were less often expressed by the students. Students with lower levels of nervousness explained this as being connected to good organization and that it did not feel like a real test. Because nervousness or anxiety can also be connected with a lack of preparation, the students were asked about their individual preparation. Some students prepared themselves in terms of content; others stated that they had only prepared a little or not at all due to good prior knowledge or lack of time.

##### 3.2.2. Pros and cons of an online test

The reasons given for the regularly reported positive experiences were the smooth assessment process, the unexpectedly realistic situation with the SPs, the positively perceived learning experience, and the feedback. The negative experiences were a result of long waiting times, personal attitudes toward SPs, and a desire for better preparation. Described as challenging was the actual online setting and its peculiar aspects regarding the unfamiliar interactions via video chat, e.g., more questions about the case history should have been asked. In respect to content, in some cases the student's own structuring of the questions while taking the case history was a challenge.

##### 3.2.3. Influence of the online format on interaction

The students identified the missing body language in themselves and in the SPs as the main difference between online and face-to-face anamnesis. The consequence of this was reported to be increased difficulty in building a relationship and difficulty in medically assessing the severity of the case. Some students developed strategies to ask more targeted questions adapted to the telemedicine setting as a way to address these limitations. Other students saw no influences.

##### 3.2.4. Suitability as a testing format versus as a learning experience

The students were unanimous in that this had been a positive learning experience. It was viewed as a good exercise, and the feedback was emphasized as positive. Even if it was not a challenge with respect to content, the format was viewed as suitable for development as a framework for anamnesis. The anamnesis station was generally perceived as an acceptable test format during the pandemic. Limitations and possibilities were discussed in regard to the assessment of practical skills. The students praised the fact that an assessment was at all possible using this format and that it was also insulated from the spread of disease. Identified as the assessment format's disadvantages were the unusual situation for all of the participants, the concerns about technical difficulties, the possibility of cheating, and the potential lack of privacy in the domestic sphere.

##### 3.2.5. Recommendations

The students commented that when teaching the topic of telemedicine not only all of the conceivable opportunities for its use should be addressed, but also aspects such as good technical equipment and good lighting. Furthermore, organizational aspects should be covered, such as having a quiet atmosphere without interruptions. The limitations in the interactions and the relevance of more pronounced facial expressions should be addressed as topics. In regard to the assessment format, the students recommended good technical equipment and focusing on the assigned task.

#### 3.3. Semi-structured interviews of simulated patients and examiners

The analysis of the interviews with the SPs and examiners showed an internal homogeneity. There were two subcategories in the category general feedback on the consultation situation: *acceptance of the online format* and *holding the consultation*. No subcategories were formed for the other categories. Attachment 2 presents the example statements and how often an aspect was mentioned by the SPs (N_SP_) and the examiners (N_P_).

The SPs and examiners showed a high acceptance of the online format due to the time and travel saved and the smooth procedure. The SPs described having the impression that both sides listened more intently because no one wanted to miss anything and that there was a more pleasant and more private atmosphere than in regular testing situations. The students, in turn, were able to adjust well to the situation and used body language themselves more intensely than usual. The examiners confirmed that the students were able to adapt well to holding a consultation in an online format.

The situation was viewed overall as *authentically real* by the SPs and the examiners, and the SPs were able to imagine such an anamnesis during an acute case. The possibilities under pandemic conditions as well as in an unusual situation were commented on. The examiners put more emphasis on the possibilities for use in terms of follow-up consultations.

Technology was rated by both sides as uncomplicated. A trial run with the technology was recommended by the SPs, and small technical challenges were discussed, such as only one person being able to speak at a time. The examiners also expressed concern about potential malfunctions of the technology.

The SPs brought up a series of *challenges*. From an actor’s point of view, starting a conversation is easier if the SP activates the video only when the time for the assessment starts. The students would then have to ask unaccustomed questions in order to better appraise the situation and the severity of the medical problem. Also, acting out pain in front of a camera is unfamiliar, requires adjustments to body language, and makes very precise descriptions of the symptoms necessary. The examiners saw no basic challenges in the evaluation. However, the changes in the students’ communication and anamnesis techniques were noticed and a revision of the checklists was suggested. From the perspective of the SPs and the examiners, the students appeared much less nervous compared to in-person assessments.

## 4. Discussion

The COVID-19 pandemic made it necessary to adapt teaching and testing formats to impart and assess competencies and to look at innovative methods for their possibilities and limitations.

Prior to the pandemic, initial experiences with online-supported OSCEs showed the positive features of saving time, lower costs, and conservation of other resources for instructors and medical schools [7], [8], [33]. Primarily technological and organizational recommendations have been derived from the experiences with online OSCEs during the pandemic [5], [16], [18]. In particular, thoroughly preparing all of the participants to use the requisite computer equipment was recommended [6], [34]. The assessment of competencies was done similarly to face-to-face settings [18]. Difficulties were reported with grading the assessment in the online format compared to the in-person format [7], [14]. Despite positive evaluations for acceptance and feasibility, the students did not desire the online format unconditionally as an alternative [20]. The reasons for this have not yet been systematically studied. Potential limitations due to physical barriers were discussed, but not verified in more detail [8], [20], [21], [26]. Our study seeks to consider the possibilities and limitations from the perspectives of all of the participants to recommend adaptations and adjustments, which could potentially lead to a higher acceptance of the assessment format [28].

We can confirm that it is possible to conduct an online OSCE station without technical or organizational problems. The scenario was perceived as realistic by the students and SPs, and the assessment method was viewed as fair. Students and examiners recognized the advantages coming from the time and travel saved.

The perception of positive learning effects, e.g., as a result of feedback [5], [21], [22], confirms the acceptance of the format, especially as an alternative during the pandemic. It was viewed as a good exercise to develop the students’ personal strategies, which can also have positive effects on self-confidence. However, due to the following limitations and necessary adjustments, the students preferred an in-person scenario more [20]. In particular, the evaluation of nonverbal communication and practical skills is often debated critically, although the assessment of communication skills within the scope of an online OSCE appears entirely feasible [8], [15]. In the interviews it was observed that the unknown consultation situation and the necessary adjustments in communicating and taking a patient's case history in a virtual format was perceived as particularly challenging by the students, although they did not view anamnesis per se as a challenge. The SPs and examiners also perceived the need to make changes in the communication with the students and felt that appropriate changes needed to be made to the roles, scripts, and the assessment checklists.

The lack of body language in the online format made it difficult, from the point of view of the students and SPs, to establish a relationship. The students perceived difficulties in making medical assessments about the case. In the interviews, some students reported having developed their own strategies for holding consultations, such as asking anamnesis questions that were adjusted to the telemedicine setting. However, they still demanded a targeted adaptation of the teaching to raise awareness of these limitations in advance. The formation of the doctor-patient relationship was much easier for the SPs, which may be a result of their professional training. They perceived the consultation as realistic, whereby the students' attempts to use body language more intensely and their altered anamnesis techniques were noticeable to them. Possible technical difficulties, such as transmission errors, fraudulent attempts (cheating) and the lack of privacy at home, e.g., through interruptions by family members, were identified as additional critical points.

## 5. Conclusion

Various aspects should be considered when further developing an online OSCE format to assess anamnesis skills as a valid alternative testing method in the future. Smooth technical implementation is possible with advance training [25], [28]. The opportunity to acquire clinical competencies and gather positive learning experiences supports the acceptance of this format. This must be considered for future uses, e.g., for off-campus students or adapting undergraduate medical education to digital processes [8], [11]. The test situation in a home environment can only be an advantage if there is an ensured private sphere [8], [11].

Changes exist in communication and anamnesis technique due to the lack of body language [7]. Necessary medical information must be asked for in a different way, as is the case in telehealth [8], [10], [11], [30]. For this reason, how students are trained must be adapted, and the scripts for the SPs and the checklists for the examiners must be revised:

- Presentation of the clinical symptoms via video chat (verbal/nonverbal)
  - different communication strategies (e.g., verbal description, clear facial expression, "showing" the problem to the camera)
- Altered/increased anamnesis questions asked by the students
  - Precise questions about the location/degree of the problem
  - Requests to have the problem described exactly
- Verbal/nonverbal communication elements
  - Clear confirmation that SP statements have been understood (e.g., verbal confirmation, nodding clearly)

## Competing interests

The authors declare that they have no competing interests. 

## Supplementary Material

Excerpts from the semi-structured interviews of the students with the frequency for the (sub)categories (NS)

Excerpts from the semi-structured interviews with the simulated patients (SP) and examiners (P) with the frequency for each category (NSP, NP)

## Figures and Tables

**Table 1:**
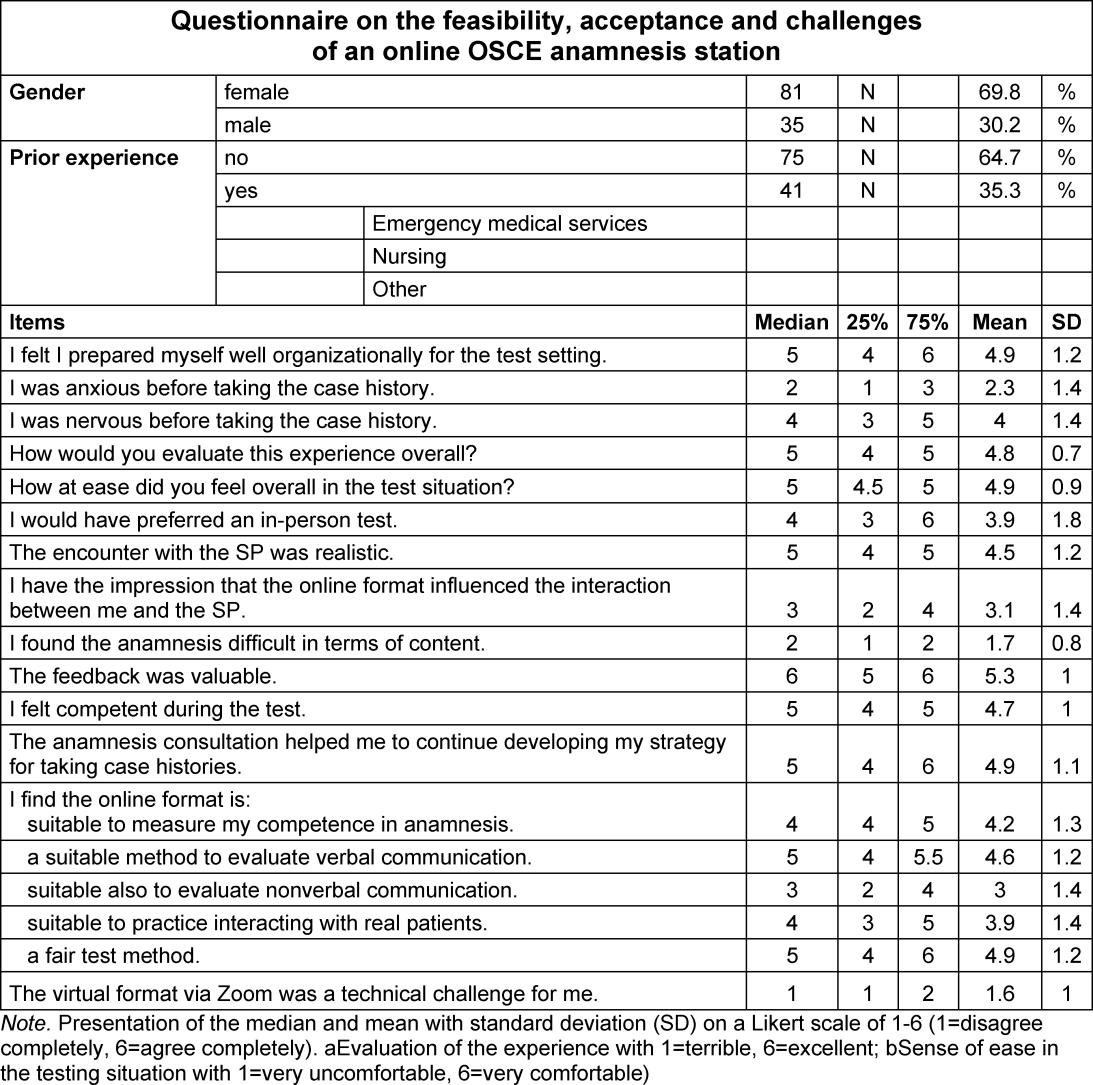
Analysis of the questionnaire responses using descriptive statistics (N=116).
