# Supplementary material for: Experience with an OSCE anamnesis station via Zoom: Feasibility, acceptance and challenges from the perspective of students, simulated patients and examiners during the COVID-19 pandemic
Source: GMS J Med Educ. 2022 Sep 15;39(4):Doc44. doi: 10.3205/zma001565 (PMC9585408; doi:10.3205/zma001565)
Supplement: Excerpts from the semi-structured interviews of the students with the frequency for the (sub)categories (NS) [file JME-39-44-s-001.pdf]

Attachment 1: *Excerpts from the semi-structured interviews of the students with the frequency for the (sub)categories (N_S)*

Excerpts from the student interviews		
Category		
**Subcategory**	N_S	**Excited vs. Anxious**
Uncertainties about online format	8	*"...did not know what to expect...."*<br>*"...how should one deal with an SP?!"*<br>*"...strange situation, will the atmosphere be different...?"*
Test situation / stress	3	*"Being at home gives me a sense of security!"*<br>*"[a] small number of people in a Zoom meeting creates a sense of security...."*
Student's own competence	4	*"I wasn't sure if I would be able to handle the clinical case?"*<br>*"I was worried about potential technical difficulties."*
Organization	4	*"I did not have any concerns, the conduction of the test was, of course, well planned."*<br>*"...[it] did not seem like a real test...."*
Student's own preparation	6 yes	*"I reviewed the basic anamnesis sample again...."*<br>*"I memorized the outline one more time."*
	4 no	*"I was doing my clerkship; I did not have any time to prepare...."*
Category		
**Subcategory**	N_S	**Pros and Cons of in-person tests**
Real (SP) situation	5 pros	*"...situation was, all in all, a little less contrived than expected."*<br>*"...friendly actors playing the patients...."*
	1 con	*"...in general, I find it difficult to engage with SPs."*
Learning experience	7	*"...positive because you are given a structure for yourself."*<br>*"...good experience because you had the chance to do it..."*<br>*"Mostly positive experience because of the feedback...."*
Organization	2 pros	*"...was pleasantly done, for instance, the preliminary remarks with the introduction."*
	3 cons	*"...[I] had to wait long and did not know what I was supposed to do during that time."*<br>*"...[I] would have liked to have had more thorough preparation."*
Online situation	12	*"...the whole thing was difficult to evaluate as a real situation."*<br>*"...the setting with non-personal contact...."*<br>*"You had to ask more questions, ...to assess the case."*<br>*"...no visual diagnosis was possible."*<br>*"...the theatrical situation is difficult to accept; in an online context even more so than face-to-face."*
Anamnesis skills	5	*"...my lack of clinical knowledge and then taking a case history...."*<br>*"...finding the transitions between the anamnesis topics."*
Category		
**Subcategory**	N_S	**Influences of the online format on interactions**
Body language	9	*"Impressions such as body odor, patient's posture, etc. are absent."*<br>*"...the nonverbal cues and body language are missing..."*<br>*"...absence of body language in one's own self."*<br>*"...no real limitations; somewhat unnatural..."*
Forming the relationship	9	*"...establishing a relationship is not so simple."*<br>*"...interpersonal communication at a distance is hard, for example, facial expressions...."*<br>*"Reliance on the patient's statements is harder than in reality."*

Attachment 1 to Herbstreit S, Benson S, Raiser C, Szalai C, Fritz A, Rademacher F, Gradl-Dietsch G. *Experience with an OSCE anamnesis station via Zoom: Feasibility, acceptance and challenges from the perspective of students, simulated patients and examiners during the COVID-19 pandemic.* GMS J Med Educ. 2022;39(4):Doc44. DOI: 10.3205/zma001565

Assessing the facts of the case	8	*"You cannot evaluate the relevance / severity of the problem."*<br>*"...assessing the disease is harder because you cannot see where the pain is."*<br>*"...you must ask more precisely...."*<br>*"...you can/must ask follow-up questions more precisely and more often; everything really depends on the words."*
Category		
**Subcategory**	N_S	**Suitability as assessment format vs. as a learning experience**
Experience as a learning opportunity	13	*"...you can practice taking a case history, find a routine."*<br>*"...things that are always forgotten are remembered better afterward."*<br>*"...recognize your own weaknesses...."*<br>*"...feedback helped me so that I will not forget some things."*<br>*"...provides a structure and gives more self-confidence...."*
Experience as a test format	19	*"Really well suited for case history taking!"*<br>*"Acceptable as a substitute, but not as the first choice...."*<br>*"Testing practical skills online would be worth a try."*<br>*"...not at all for practical skills!"*<br>*"...that a test can even take place!"*<br>*"...was a more relaxed setting, more so than in a room. I was less nervous!"*
"Spatial" setting	5 pros	*"...saved us from having to travel...."*<br>*"...separate from the infection numbers."*<br>*"Certainty in terms of scheduling..."*
	8 cons	*"It was new for everyone–including the examiners–everyone had to get their bearings."*<br>*"Situation wasn't very real...."*<br>*"Technical difficulties could occur."*<br>*"Private sphere at home is maybe more limited...."*<br>*"...maybe unfair because you can cheat!?"*
Category		
**Subcategory**	N_S	**Recommendations**
Technical challenges	17	*"...no problems, I had also set up an alternative."*<br>*"...I would always recommend using a large computer screen...."*<br>*"...choose a device with camera and microphone...."*<br>*"...tell yourself it is about testing your own knowledge and ability."*<br>*"...concentrate on the consultation and not on the test."*<br>*"...to feel as if you are not being observed...."*
Challenges / potentials of telemedicine	14	*"...use in patient training sessions, for instance."*<br>*"...pay attention to the details like lighting, camera angle, etc."*<br>*"Be prepared to use aids, for example, to illustrate something or be ready to be shown something as a drawing."*<br>*"...be without interruptions, this goes for doctor and patient...."*<br>*"Identify the limitations of the communication, raise awareness of them...."*<br>*"...communicate the importance of facial expressions and use them very deliberately...."*

SP=simulated patient
