# Supplementary material for: Experience with an OSCE anamnesis station via Zoom: Feasibility, acceptance and challenges from the perspective of students, simulated patients and examiners during the COVID-19 pandemic
Source: GMS J Med Educ. 2022 Sep 15;39(4):Doc44. doi: 10.3205/zma001565 (PMC9585408; doi:10.3205/zma001565)
Supplement: Excerpts from the semi-structured interviews with the simulated patients (SP) and examiners (P) with the frequency for each category (NSP, NP) [file JME-39-44-s-002.pdf]

Attachment 2: *Excerpts from the semi-structured interviews with the simulated patients (SP) and examiners (P) with the frequency for each category (N_{SP}, N_P)*

	**Excerpts from SP interviews**	**Excerpts from examiner interviews**
**Category**<br>Subcategory	**General feedback on the patient consultation**	
Acceptance of the online format	(N_{SP}=6)<br>- *"... [I] felt it was very good."*<br>- *"It is a great thing, I have to say."*<br>- *"I was able to get into the situation really well...it is a good alternative."*<br>- *"It was good, it was really relaxed...."*	(N_P=4)<br>- *"I find it good that I can do this from my office...."*<br>- *"...I could work this into my schedule from here on out!"*<br>- *"It took place without any problems!"*
Holding the consultation	(N_{SP}=8)<br>- *"...overall, I am astonished how well the students were able to adjust to the situation, how all of them really held a personal conversation."*<br>- *"...I had the feeling that we–although we were not sitting in the room–were nonetheless very close to one another."*<br>- *"...you listened more closely because you didn't want to miss anything..., paid more attention to nodding and such things...."*<br>- *"I almost have the feeling that you speak more attentively with each other ... that it is somehow a little more personal."*<br>- *"...forming a relationship really does happen! You really get to know each other now."*	(N_P=5)<br>- *"I am amazed how well the students adapted to the situation."*<br>- *"Some students were not sure how they should ask questions since they were unable to see the patient's injury."*<br>- *"Now and then a student did not register that it was an online consultation...."*<br>- *"They really made an effort to question the SPs in a way that got at the heart of the issue but without being able to see anything...."*
**Category**	**Evaluating the authenticity**	
	(N_{SP}=6)<br>- *"...that you can get a diagnosis at home ... I don't think that's bad."*<br>- *"It is, of course, at the moment a little funny to think: OK, I'll just have a chat to talk about my foot...but who knows what we have to prepare ourselves for."*<br>- *"...at some point I felt I was being responded to and had the impression that, yeah, it is possible to take the initial case history this way."*<br>-	(N_P=3)<br>- *"Regarding the students who understood that this is a video appointment, they really made it seem real...!"*<br>- *"...I can imagine that I can do this with a patient I know, but for the very first appointment...?"*
**Category**	**Identifying the problems**	
	(N_{SP}=5)<br>- *"...works super with the iPad, which is easy to use; this is not at all a problem."*<br>- *"...something that is really important here with the acting via video is that only one person can speak at a time."*<br>- *"...perhaps you should do a soundcheck of some sort and see if the volume needs to be turned up...."*	(N_P=5)<br>- *"Technically everything went without a glitch...!"*<br>- *"I thought it was good that I could turn my camera and microphone off so that the others were alone with each other...."*<br>- *"...you can only hope there is no technical disruption...!"*

Attachment 2 to Herbstreit S, Benson S, Raiser C, Szalai C, Fritz A, Rademacher F, Gradl-Dietsch G. *Experience with an OSCE anamnesis station via Zoom: Feasibility, acceptance and challenges from the perspective of students, simulated patients and examiners during the COVID-19 pandemic.* GMS J Med Educ. 2022;39(4):Doc44. DOI: 10.3205/zma001565

**Category**	**Did the students conduct themselves differently in the virtual OSCE?**	
	$(N_{SP}=7)$	$(N_P=7)$
	- *"Not everything about how the students went on to ask questions was explicit in the script ... I then made things up."*<br>- *"As an SP, you have to describe your complaints differently than usual because they can't interpret the body language so well...."*<br>- *"...you had to answer some unfamiliar and detailed questions, that is not usually the case."*<br>- *"...naturally you pay more attention to how you have to act differently to show the medical complaint."*	- *"The evaluation was actually the same as in the face-to-face test."*<br>- *"The students were all well prepared; I mostly gave positive feedback!"*<br>- *"The additional questions that the students had to ask due to the online context were not on the checklist and time got tight in some cases...."*<br>- *"...There is no explicit training for things like this!"*
**Category**	**Student nervousness**	
	$(N_{SP}=6)$	$(N_P=6)$
	- *"...the [students] are less nervous. Perhaps because there are not so many people in the room and the instructor is not visible."*<br>- *"...maybe even a bit more relaxed because they are at home."*<br>- *"...I have the feeling those who are less nervous...feel more comfortable at home. ...You make a fool of yourself all alone and not in front of your colleagues."*<br>- *"Yes, they are a bit more relaxed.... But they are still very attentive, very present and concentrated."*	- *"...they appear very relaxed, and even sometimes really self-assured!"*<br>- *"Some of them were much less nervous than otherwise...."*<br>- *"You could see that a few of them were somewhat nervous, but they were otherwise well prepared."*

Attachment 2 to Herbstreit S, Benson S, Raiser C, Szalai C, Fritz A, Rademacher F, Gradl-Dietsch G. *Experience with an OSCE anamnesis station via Zoom: Feasibility, acceptance and challenges from the perspective of students, simulated patients and examiners during the COVID-19 pandemic.* GMS J Med Educ. 2022;39(4):Doc44. DOI: 10.3205/zma001565
